# First confirmation by PCR of Jaagsiekte sheep retrovirus in Ireland and prevalence of ovine pulmonary adenocarcinoma in adult sheep at slaughter

**DOI:** 10.1186/s13620-017-0111-z

**Published:** 2017-12-19

**Authors:** Alison Marie Lee, Alan Wolfe, Joseph P. Cassidy, Locksley L. McV. Messam, John P. Moriarty, Ronan O’Neill, Claire Fahy, Emily Connaghan, Chris Cousens, Mark P. Dagleish, Maire C. McElroy

**Affiliations:** 1 School of Veterinary Medicine, University College Dublin, Belfield, Dublin 4, D04 W6F6 Ireland; 2 Department of Agriculture, Food and the Marine Laboratories, Backweston Laboratory Campus, Celbridge, Co. Kildare W23 X3PH Ireland; 3 Moredun Research Institute, Pentlands Science Park, Bush Loan, Penicuik, Midlothian EH26 0PZ Scotland, UK

**Keywords:** Jaagsiekte, Jsrv, Jaagsiekte sheep retrovirus, Opa, Ovine pulmonary adenocarcinoma, Diagnostics, Neoplasia, Retrovirus, Prevalence, Ireland

## Abstract

**Background:**

Ovine pulmonary adenocarcinoma (OPA), caused by Jaagsiekte sheep retrovirus (JSRV), is characterised by the development of invariably fatal lung tumours primarily in adult sheep. High infection rates and disease prevalence can develop during initial infection of flocks, leading to on-farm economic losses and animal welfare issues in sheep with advanced disease. The disease has been reported in Ireland and is notifiable, but the presence of JSRV has never been confirmed using molecular methods in this country. Additionally, due to the difficulties in ante-mortem diagnosis (especially of latently-infected animals, or those in the very early stages of disease), accurate information regarding national prevalence and distribution is unavailable. This study aimed to confirm the presence of JSRV in Ireland and to obtain estimates regarding prevalence and distribution by means of an abattoir survey utilising gross examination, histopathology, JSRV-specific reverse transcriptase polymerase chain reaction (RT-PCR) and SU protein specific immunohistochemistry (IHC) to examine the lungs of adult sheep.

**Results:**

Lungs from 1911 adult sheep were examined macroscopically in the abattoir and 369 were removed for further testing due to the presence of gross lesions of any kind. All 369 were subject to histopathology and RT-PCR, and 46 to IHC. Thirty-one lungs (31/1911, 1.6%) were positive for JSRV by RT-PCR and/or IHC but only ten cases of OPA were confirmed (10/1911, 0.5%) Four lung tumours not associated with JSRV were also identified. JSRV-positive sheep tended to cluster within the same flocks, and JSRV-positive sheep were identified in the counties of Donegal, Kerry, Kilkenny, Offaly, Tipperary, Waterford and Wicklow.

**Conclusions:**

The presence of JSRV has been confirmed in the Republic of Ireland for the first time using molecular methods (PCR) and IHC. In addition, an estimate of OPA prevalence in sheep at slaughter and information regarding distribution of JSRV infection has been obtained. The prevalence estimate appears similar to that of the United Kingdom (UK). Results also indicate that the virus has a diverse geographical distribution throughout Ireland. These data highlights the need for further research to establish national control and monitoring strategies.

**Electronic supplementary material:**

The online version of this article (10.1186/s13620-017-0111-z) contains supplementary material, which is available to authorized users.

## Background

Ovine pulmonary adenocarcinoma (OPA) is a contagious, neoplastic lung disease of sheep caused by Jaagsiekte Sheep Retrovirus (JSRV). It is found worldwide, with the exception of Australia, New Zealand, the Falkland Islands and Iceland [1]. It received international veterinary attention after an outbreak in Iceland in the 1930s in which one-third of the sheep population was affected and the disease was controlled by extensive culling [1]. JSRV is an oncogenic retrovirus which causes lung epithelial cells (type II pneumocytes and club cells) to form invasive bronchiolo-alveolar carcinomas [2, 3]. Clinical signs in animals with large tumours include dyspnoea, tachypnoea, exercise intolerance, nasal discharge, coughing, loss of condition and, in a proportion of cases, the production of copious amounts of lung fluid [1]. The virus is spread via aerosol and probably via milk and colostrum also [1, 4] with lambs being highly susceptible to infection while adults are less so [5, 6]. After initial infection, it is thought the virus may persist for several years in circulating leukocytes before invading pneumocytes and club cells to cause neoplastic transformation [5]. Clinical disease usually occurs in adult animals (two to four years old) [1]. After initial entry into a flock, JSRV may remain unnoticed for some time even though many animals may be infected, because the disease can remain subclinical throughout their commercial lifespan without the development of neoplastic lesions [5]. JSRV does not induce a humoral immune response in infected animals [7] and this is thought to be because endogenous JSRV-related retroviruses (enJSRVs), present in the sheep genome, may induce a state of immunotolerance [8]. Therefore serological testing is not available. Ante-mortem diagnosis using other methods (PCR on blood/bronchoalveolar lavage [BAL] fluid, thoracic ultrasound) lacks the sensitivity and specificity needed to reliably diagnose latently infected animals or the early sub-clinical stages of OPA. Sensitivity of PCR on a single blood sample is only 11% [9], and hence this test may be useful for flock screening but not individual animal diagnosis. PCR on fluid collected by BAL has a sensitivity of 89% [10] but the technique is cumbersome to perform in the field. Thoracic ultrasound may identify lesions as small as 1-2 cm in diameter if they involve the pleural surface, but may miss smaller nodules or lesions deep within the pulmonary parenchyma [11]. Hence definitive diagnosis is usually made by post-mortem examination [12]. Confirmatory histological examination of lung lesions is essential for a diagnosis of OPA [13]. IHC may also be required to identify small lesions or those obscured by concurrent pathology [1, 12], but is not, to the author’s knowledge, routinely used as commercial test. A discussion of OPA control measures is beyond the scope of this work, but are outlined by Scott et al., 2013 [12].

Traditionally, individual sheep are considered to be of relatively low economic value, but the industry was worth €244.5 million^1^ to the Irish economy in 2015 (4% of the total worth of the agricultural sector) [14]. In June 2016 there were 5.18 million sheep in Ireland (2.6 million of which were breeding sheep), a 0.7% increase on 2015 [15]. OPA was first reported in Ireland in the mid-1980s [16]. However, little information is available on the regional prevalence or distribution of OPA and there is no information on JSRV infection because molecular methods to detect JSRV have not been reported in Ireland. Table 1 provides the number of OPA cases diagnosed in Ireland over the last five years, based on post-mortem examination of sheep submitted to the veterinary diagnostic laboratory at University College Dublin (UCD), the Irish Department of Agriculture, Food and the Marine (DAFM) Veterinary Laboratory Service and the Agri-Food and Biosciences Institute (AFBI). The latter provides veterinary diagnostic services in Northern Ireland. However there is no information on prevalence and distribution across the island. Historically, higher percentages of sheep with OPA have been recorded in Northern Ireland compared to the Republic of Ireland [17].

**Table 1:** OPA cases diagnosed post-mortem at University College Dublin (UCD), Department of Agriculture, Food and the Marine (DAFM) and the Agri-Food and Biosciences Institute (AFBI), 2010 to 2016 (Cloak B, UCD; McElroy M, DAFM; Guelbenz, M, AFBI; personal communications)

	UCD			DAFM			AFBI		
	No. OPA+ sheep at PM	No. PMs sheep >6 months	% OPA +	No. OPA+ sheep at PM	No. PMs sheep >6 months	% OPA +	No. OPA+ sheep at PM	No. PMs sheep >6 months	% OPA +
2016	7	16	43.8	9	794	1.1	27	237	11.4
2015	2	18	11.1	8	431	1.9	21	250	8.4
2014	1	13	7.7	9	394	2.3	25	246	10.2
2013	1	13	7.7	5	514	1	23	332	6.9
2012	0	14	0	5	437	1.1	16	406	3.9
2011	1	3	33	6	437	1.4	17	268	6.3
2010	1	11	9.1	1	362	0.3	16	302	5.3

Confirmation of the disease and estimation of prevalence in other countries has generally been carried out via abattoir surveys, due to the absence of effective ante-mortem diagnostic tests [18–21]. A recent abattoir-based survey in the UK, using macroscopic, histologic, and immunohistochemical techniques, diagnosed OPA in 0.9% of adult sheep [18]. As spontaneously arising bronchiolo-alveolar adenocarcinomas not associated with JSRV have been reported in sheep [22, 23], it is important to definitively establish if lung tumours diagnosed in sheep in Ireland are due to JSRV infection. Also, as OPA is a notifiable disease, it is important to estimate national prevalence and geographical distribution in Ireland as this may assist farmers in sourcing JSRV-free replacements to maintain uninfected flocks or replace infected flocks. A PCR test has been recently made available by DAFM, and may be useful for Irish flock health monitoring. Given the contribution of the sheep sector to the Irish agri-food industry, knowledge of the prevalence and distribution of any endemic diseases is essential for monitoring, control and the design of health schemes.

This aim of this study was to definitively confirm the presence of JSRV infection and OPA in the Republic of Ireland, and to obtain preliminary information regarding proportion of animals affected and disease and infection distribution by means of an abattoir survey and subsequent molecular tests.

## Results

### Sheep numbers and geographical distribution

Lungs of 1911 sheep aged over one year from 18 of the 26 counties in the Republic of Ireland were examined visually and palpated for gross lesions of any type. Grossly-visible and/or palpable lesions were found in lungs from 369 sheep (19.3%) which were then subjected to further examination. These sheep originated from 76 (58%) of the 127 flocks examined and from 17 of the 18 counties. The number of sheep and flocks per county examined in this study varied widely (Table 2).

**Table 2:** Numbers of sheep >1 year old grossly examined and sampled, and numbers of flocks examined and sampled, based on their country of origin

County	Total number of sheep examined	Total number of sheep sampled	Total number of flocks examined	Total number of flocks sampled from
Carlow	22	2	2	1
Cork	220	36	14	9
Donegal	168	51	4	3
Dublin	7	2	1	1
Galway	15	1	4	1
Kerry	490	90	34	21
Kildare	111	21	15	8
Kilkenny	55	21	1	1
Laois	21	1	6	1
Mayo	105	6	11	4
Meath	120	24	7	5
Offaly	42	9	3	2
Roscommon	68	11	2	2
Sligo	6	0	1	0
Tipperary	179	25	4	3
Waterford	48	15	5	4
Westmeath	7	2	2	2
Wicklow	227	52	11	8

### Macroscopic examination

**Fig. 1:**
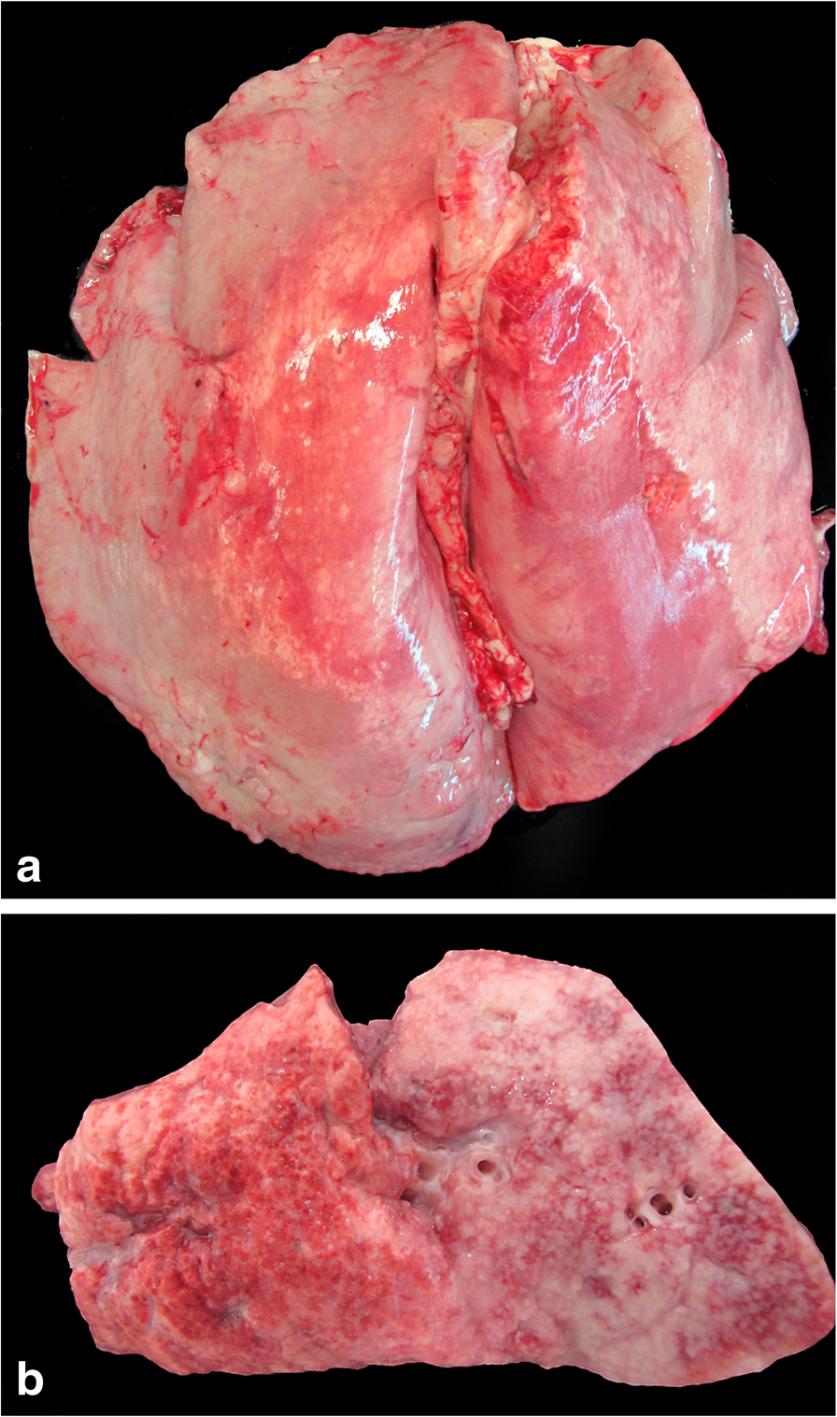
**a** Set of lungs with ovine pulmonary adenocarcinoma [OPA], with lesions consistent with those of classical OPA. Bilateral, extensive, ventral areas of greyish discolouration, **b** OPA-positive lung tissue as seen on cut surface. Area of normal lung (salmon-pink) on the left. Area of OPA (grey-tan, fatty, consolidated) to the right

**Fig. 2:**
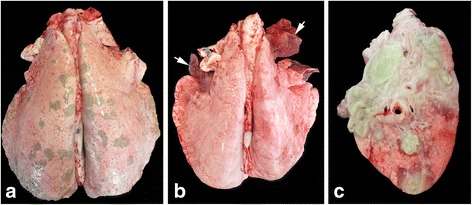
**a** Set of lungs affected by lungworm. Multifocal, granular, grey-green subpleural granulomas bilaterally affecting the dorsocaudal lung lobes, **b** Set of lungs affected by subacute bronchopneumonia. Here, right and left cranial lobes and right middle lobe are dark red and consolidated (arrows). The cranial part of the left cranial lobe is missing (torn off when removed from thoracic cavity due to pleural adhesions), **c** Multifocal-coalescing lung abscess seen in cross-section. There are multiple cavities containing yellow, viscous material (pus) surrounded by fibrous connective tissue capsules

Eighteen lungs were classed as ‘Macroscopically-Suspect OPA’ (MSO) based on gross examination (Table 3) which represents 0.1% of all the 1911 sets of lungs inspected, and 4.9% of the lungs with gross lesions of any kind. The numbers of other gross lesions identified are given in Additional file 1: Table S1. An example of MSO is shown in Fig. 1a and b. Other typical macroscopic findings, such as subpleural parasitic granulomas, cranioventral consolidation and pulmonary abscessation, are shown in Fig. 2 a, b and c, respectively.

**Table 3:** Criteria used for the classification of lungs based on macroscopic examination after removal from the abattoir

Macroscopic Lesion Category	Description
Abscess(es)	Single-multiple, variably-sized, well-demarcated, round nodules with fibrous capsule and central cavity filled with white/green/yellow creamy material (pus)
Cranioventral consolidation	Ventral aspects of cranial +/− middle lung lobes dark red-purple in colour, well-demarcated, firm on palpation
Discolouration	Any region of discolouration distinct from others described in classification system
Fibrosis	Firm, pale, fibrous scar tissue
Focal firm nodule	Focal-multifocal, variably-sized, well-demarcated, firm, homogenously solid, grey nodule(s) within parenchyma
Mineralisation	Mineralised foci not consistent with parasitic granulomas
Other	Lesions not consistent with others listed
Subpleural parasitic granulomas	Multifocal pale tan/green well-demarcated, irregularly-circular slightly raised foci, up to2cm diameter, on dorsal aspect of the caudal lung lobes, +/− mineralisation
Macroscopic Suspect OPA (MSO)	Locally-extensive, well-demarcated, consolidated, heavy, oedematous, grey-purple areas
Uncollapsed	Diffusely uncollapsed

### Histological examination

**Fig. 3:**
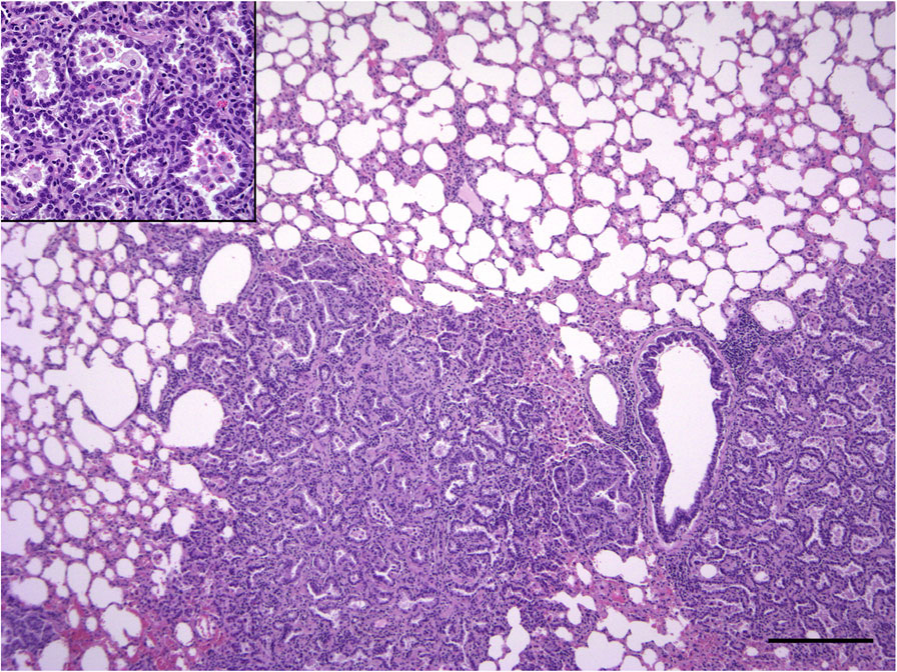
Two coalescing foci of ovine pulmonary adenocarcinoma present in the right ventral area of the image. Neoplasm is well-demarcated to infiltrative with an acinar growth pattern supported by a fibrous stroma. Inset (top left): Clusters of macrophages present in the acinar spaces formed by cuboidal tumour cells. Haematoxylin and eosin. Bar = 250 μm

All 369 lungs with gross lesions of any kind were examined histologically and the lesions were categorised as described in Table 4. Lung sections from 12 animals were defined as ‘Histologically-Suspect OPA’ (HSO), but only two of these had been classed as MSO. An example of OPA-positive lung tissue is shown in Fig. 3. Numbers of other histologic lesions identified are given in Additional file 1: Table S2. Twelve macroscopically-normal control lungs were also examined histologically; five had interstitial thickening, while the others had no significant lesions. Inter-observer agreement between the three pathologists who assessed the histological lesions was ‘substantial’, as determined by the Kappa squared test (K = 0.8, 95% confidence interval [CI] = 0.7–0.9), indicating that the lesion classification system adopted was consistent [24].

**Table 4:** Criteria used for the classification of lungs based on histologic examination

Histological lesion category	Description
Abscess	Thick fibrous capsule (or part thereof) surrounding viable +/− degenerate neutrophils +/− central liquefactive necrosis
Bronchopneumonia	Alveolar and bronchial/bronchiolar lumina filled with admixed neutrophils, macrophages and lymphocytes, +/− type 2 pneumocyte hyperplasia, bronchiolitis obliterans, oedema, bacterial colonies, regions of fibrosis
Interstitial thickening	Extensive-diffuse, mild-marked thickening of the alveolar septa due to smooth muscle hypertrophy +/− fibroblasts.
Histologically Suspect OPA (HSO)	Single-multifocal, well-demarcated-infiltrative tumours with papillary/lepidic/acinar growth patterns consisting of cuboidal-columnar cells supported by a fine fibrous stroma and surrounded by macrophage infiltration.
Parasitic infestation	One or more of the following: nematode eggs/larvae, eosinophilic granulomas, eosinophil-rich interstitial and intra-alveolar infiltrates.
Other	Pathological changes not falling into any of the other categories described.
Normal	No pathological changes

### RT-PCR for JSRV

All 369 sheep with gross lesions were subjected to JSRV RT-PCR. Thirty were positive, with CT-values <38. Of these JSRV RT-PCR-positive animals, four had been classified as MSO, five as HSO and two as both MSO and HSO. Thus 19 samples were JSRV RT-PCR-positive with no gross or histological evidence of OPA. In addition, five samples classified as HSO were JSRV RT-PCR-negative.

### IHC for OPA

**Fig. 4:**
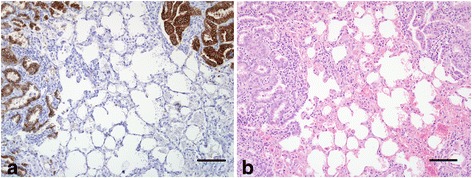
**a** Expression of Jaagsiekte sheep retrovirus SU protein in cytoplasm of tumour cells of ovine pulmonary adenocarcinoma. **b** Negative control. Bar = 100 μm

Forty-six lung tissue sections from all animals that were MSO, HSO and/or RT-PCR-positive were subjected to IHC (tissue from one MSO-positive animal, Sheep 46, was unavailable for IHC). Ten sheep were positive and three, eight and nine of these had been categorised as MSO, HSO or RT-PCR-positive, respectively. Histological sections from the two sheep found to be IHC+, RT-PCR+, and HSO- were reviewed and in one, severe bronchopneumonia masked OPA lesions. In the other, early-stage OPA lesions were present but obscured by extensive, severe parasitic lesions. An example of IHC+ lung tissue control is shown in Fig. 4.

**Fig. 5:**
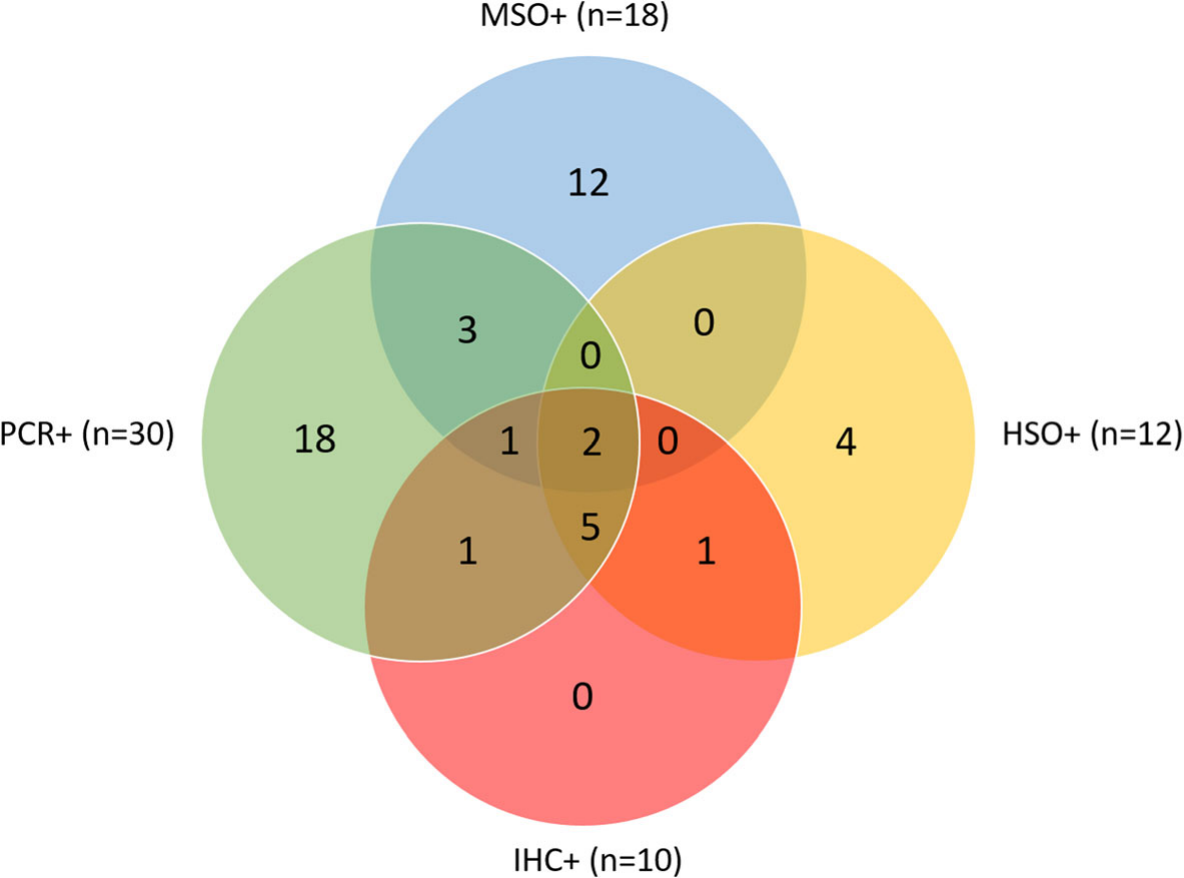
Venn diagram showing the distribution of the results of the various tests for ovine pulmonary adenocarcinoma between sheep, including macroscopic examination, histologic examination, immunohistochemistry (IHC), and polymerase chain reaction (PCR). HSO = histologically-suspect OPA, MSO = macroscopic- suspect OPA

Table 5 and the Venn diagram shown in Fig. 5 summarise the results for sheep that were found to be OPA-positive by one or more of the examination methods employed. Overall, 11 of the 18 animals classified as MSO were negative for OPA and JSRV by histological examination, RT-PCR and IHC, while one was negative by histological examination and RT-PCR only, as tissue was unavailable for IHC. Only two of the 18 had OPA confirmed histologically (Sheep 1 and 12) and one other had OPA confirmed only after IHC (Sheep 5). Six of these 18 MSO sheep had virus detected by RT-PCR, three of which had tumours histologically or after IHC (Sheep 1, 5 and 12). Twelve animals had histological lesions found on initial examination that were consistent with OPA (Sheep 1, 7, 10, 12, 16, 18, 22, 23, 29, 30, 32, 35) and a further two more had histological OPA lesions revealed by IHC only (Sheep 4 and 5). Ten of these 14 neoplasms were found to be associated with JSRV (ten were IHC-positive and nine of the 10 were RT-PCR-positive also). The remaining four neoplasms were JSRV negative by RT-PCR and IHC and therefore represent bronchiolo-alveolar adenocarcinomas not related to JSRV (Sheep 16, 22, 23, 29). Thirty-one animals were confirmed to have JSRV infection in lung tissue, either by RT-PCR alone (*n* = 21), IHC alone (*n* = 1), or RT-PCR and IHC (*n* = 9). Only two animals were positive for JSRV/OPA on macroscopic, histological, RT-PCR and IHC examination (Sheep 1 and 12).

**Table 5:** Details of macroscopically/histologically suspect OPA and JSRV-positive animals on PCR/IHC

Sheep ID	Flock ID	County of origin	Macroscopic diagnosis	Histologic diagnosis	PCR +/−	IHC +/−
1	A	Donegal	MSO	HSO	+	+
2	A	Donegal	F, P	P	+	–
3	A	Donegal	A	A	+	–
4	A	Donegal	D, P	IT	+	+
5	A	Donegal	CC, P, MSO	B	+	+
6	A	Donegal	CC, MSO	B	+	–
7	A	Donegal	A, FFN, F, P	HSO	+	+
8	A	Donegal	A, F	A	+	–
9	A	Donegal	CC, P	B	+	–
10	A	Donegal	FFN, F, P	HSO, B	+	+
11	A	Donegal	CC	B	+	–
12	A	Donegal	MSO	HSO, B	+	+
13	A	Donegal	CC	IT	+	–
14	A	Donegal	CC, P	B	+	–
15	A	Donegal	MSO	B	+	–
16	B	Kerry	FFN	HSO	–	–
17	B	Kerry	P	P	+	–
18	C	Kilkenny	D	HSO	+	+
19	C	Kilkenny	P	P	+	–
20	C	Kilkenny	P	P	+	–
21	C	Kilkenny	CC, P	IT	+	–
22	D	Mayo	FFN	HSO	–	–
23	E	Meath	D	HSO	–	–
24	F	Offaly	A, P	A	+	–
25	F	Offaly	P	P	+	–
26	G	Tipperary	M	IT	+	–
27	H	Tipperary	MSO	IT	+	–
28	H	Tipperary	CC	IT	+	–
29	I	Waterford	A	HSO, A	–	–
30	J	Waterford	FFN, F, M	HSO, A	+	+
31	J	Waterford	P	N	+	–
32	K	Wicklow	A	HSO, B	+	+
33	K	Wicklow	A, P	A	+	–
34	K	Wicklow	CC, D	B	+	–
35	L	Wicklow	A	HSO	–	+
36	K	Wicklow	MSO	B	–	–
37	C	Kilkenny	MSO, P	B, P	–	–
38	C	Kilkenny	MSO	B	–	–
39	K	Wicklow	MSO	B	–	–
40	A	Donegal	MSO, P	B	–	–
41	A	Donegal	MSO	B	–	–
42	A	Donegal	P, MSO	IT	–	–
43	M	Tipperary	A, F, MSO	A, B	–	–
44	N	Mayo	P, MSO	B	–	–
45	B	Kerry	A, MSO	IT	–	–
46	B	Kerry	MSO	B	–	ND
47	A	Donegal	CC, MSO	B	–	–

*A* Abscess, *B* Bronchopneumonia, *CC* Cranio-ventral consolidation, *D* Discolouration, *F* Fibrosis, *FFN* Focal firm nodule, *HSO* Histologically-suspect OPA, *IT* Interstitial thickening, *M* Mineralisation, *MSO* Macroscopically-suspect OPA, *ND* Not done, *P* Parasitic

In summary, JSRV infection was detected in 1.6% (31/1911, 95% CI: 1.1–2.3%) of the adult sheep in this survey (8.4% [31/369, 95% CI: 5.4–11.5%] of the samples collected). JSRV-associated neoplasms (OPA) were identified in 0.5% (10/1911, 95% CI: 0.25–0.96) of the adult sheep in this survey (2.7% [10/369, 95% CI: 0.7–4.7] of the samples collected). Thus the estimated prevalence of JSRV infection and OPA in adult sheep are 1.6% and 0.5% respectively. Additionally, bronchiolo-alveolar tumours of unknown origin were found in 0.2% (4/1911, 95% CI: 0.06–0.5%) of the animals in this survey (1.1% [4/369, 95% CI: 0.3–2.8] of the samples collected).

## Methods

### Lung collection

Ten visits to a DAFM-approved abattoir in Co. Kildare, Ireland were carried out between September and November 2015. This coincided with a peak time for culling adult sheep. The lungs of 1911 sheep (primarily ewes and a small number of rams), all over one year old, from 127 different flocks were examined post-mortem, both visually and by palpation, for the presence of lung lesions of any type. This was carried out by two to three persons on each visit, including A. M. Lee (veterinary anatomical pathology resident; present at every visit) accompanied by one or two experienced DAFM veterinarians. Lungs with macroscopic lesions of any type were collected and transported within 1–5 h to The DAFM Central Veterinary Research Laboratory, County Kildare for further examination (described below), which was performed on the same day. In addition, 12 grossly normal lungs were sampled for comparative purposes. The county of origin and flock number of each sheep examined was obtained from the abattoir’s computer system but it was not possible to identify the sex or exact age. No animals were slaughtered specifically for the purpose of this study.

### Macroscopic examination

Gross lesions present in each pair of lungs were recorded diagrammatically and categorised as described in Table 3. Definitions and categories of lesions were based on common gross pathological changes of sheep lungs [25]. Two tissue samples, 10mm^3^ and 2mm^3^ respectively, were taken from the main visible/palpable lesion of interest from each set of lungs for histological examination and RT-PCR analysis, respectively. Samples from the 12 grossly-normal lungs were taken consistently from the dorsal aspect of the right caudal lung lobe for identical histological examination and RT-PCR analysis.

### Histological examination

Tissue samples for histopathology were fixed in 10% neutral buffered formalin, dehydrated through graded alcohols, embedded in paraffin wax, sectioned (5 μm-thick) and stained with haematoxylin and eosin (H&E). Sections were examined by light microscopy (Nikon Eclipse E600) and assigned to one or more of seven categories (Table 4) based on commonly reported histological lesions in sheep lungs [25]. Sections were examined independently by three pathologists without knowledge of the gross appearance of the lesion. Inter-observer agreement in the categorisation process was assessed by the Kappa statistic using an online statistical software programme [26].

### RT-PCR for JSRV

Real-time RT-PCR was used to detect JSRV in samples of lung tissue along with appropriate negative control (RNase-free water) and positive control (Nucleic acids [NA] extracted from lung fluid from an OPA-positive sheep from Scotland) preparations. Briefly, NA was extracted from lung tissue using the MagNA Pure LC Total Nucleic Acid Isolation Kit or MagNAPure 96 automated extraction protocol (Roche Diagnostics). Quantitative RT-PCR was performed using a Stratagene Mx3000/3005 thermal cycler (Stratagene, Agilent Technologies) as follows: a PCR cocktail containing 5 μL TaqMan® Fast Virus 1-Step Master Mix (Biosciences Cat. No. 4444434), 0.5 μL JSRV P1 F primer (10pM/uL, SigmaGenosys), 0.5 μL JSRV P6 R primer (10pM/uL, SigmaGenosys), 0.5 μL JSRV JS-T FAM probe (2pM/uL, SigmaGenosys) and 8.5 μL distilled water was added to each well of a QPCR plate. 5 μL of sample NA/control sample was added to the wells in duplicate. Cycle conditions were 50 °C for 30 min, followed by 95 °C for 15 min, then 45 consecutive cycles of 94 °C for 30 s and 58 °C for one minute (during which time data analysis and real-time analysis was enabled). Test samples with a cycle threshold (CT) of <38 were considered JSRV-positive. Primers and probe sequences [27, 28] are provided in Table 6.

**Table 6:** Jaagsiekte Sheep Retrovirus RT-PCR primer and probe sequences

JSRV P1	5’ TGGGAGCTCTTTGGCAAAAGCC-3′
JSRV P6	5’-TGATATTTCTGTGAAGCAGTGCC-3′
JSRV JST FAM probe	5’-FAM-AGCAAACATGGCARCCTTAAGAGCTTTCAAAA-3′

### Immunohistochemistry

Any lung sample categorised as ‘MSO’ on gross examination, ‘HSO’ on histological examination, and/or any that yielded a positive RT-PCR result (total: *n* = 46) were subjected to IHC using a primary antibody against the SU sub-unit of the envelope protein of JSRV, as described previously [29]. Tissue from one sheep that was MSO-positive (Sheep 46) was unavailable for IHC. Briefly, sections were dewaxed and rehydrated to water. Endogenous peroxidase activity was blocked by incubation in 3% H_2_O_2_ in methanol (*v*/v) for 20 min, then washed in running tap water for five minutes. Slides were then placed in citrate solution, pH 6.0, at 121 °C for 10 min for antigen retrieval, washed, and non-specific antibody binding was blocked by incubation with 25% normal goat serum (Vector S1000) diluted in phosphate buffered saline containing 0.05% Tween_20_ (PBST) for 30 min prior to incubation overnight with the primary antibody (mouse anti-SU protein, diluted 1/250 in PBST). After washing, bound primary antibody was visualised with the DAKO EnVision™ + System (goat anti-mouse, DAKO) as per the manufacturers’ instructions using 3, 3′-diaminobenzidine as a substrate (Sigma-Aldrich). Sections were counterstained with Haematoxylin Z, blued in Scots tap-water substitute, dehydrated, cleared and mounted. Slides were washed in PBST between procedures unless otherwise stated.

## Discussion

This study confirms the presence of JSRV-infected sheep in Ireland and demonstrates that the virus may be isolated from sheep that do not have gross or histologic evidence of OPA. Infected sheep were identified in seven counties, with Donegal having the highest percentage of cases. Of the 1911 adult sheep that were examined in this survey (comprising 0.07% of the breeding sheep of the national flock in 2015 [15]), 1.6% were infected with JSRV and 0.5% had OPA. This is similar to that of a recent abattoir survey carried out in the UK where 0.9% of 3385 adult sheep were found to be OPA positive [18]. However, in contrast to the current study, RT-PCR for JSRV on lung tissue was not used in the UK study. This may have resulted in a higher prevalence of JSRV infection than the number found with OPA. Importantly, the present study also identified cases of non-JSRV-associated bronchiolo-alveolar adenocarcinoma, highlighting the potential misdiagnosis of early-stage OPA when molecular testing for JSRV or specific IHC is not performed.

It is unlikely that the prevalence estimate identified in this single pilot study represents the true prevalence in the national flock, due to the various factors common to many animal disease surveys. This study used abattoir material, and although abattoirs are convenient sources of animal tissue samples, their primary function is to slaughter clinically healthy animals for human consumption. This may lead to biases in the data collected from this source, with some factors leading to possible over-estimation of the disease prevalence, and other factors leading to possible under-estimation of disease prevalence [30]. One source of bias is the exclusion of clinically diseased animals which inevitably distorts true disease prevalence estimations. Additionally, the proportion of sheep examined in this study from each county was not fully representative of the national distribution of sheep, and no sheep were examined from eight counties in the Republic of Ireland (Cavan, Clare, Leitrim, Limerick, Longford, Louth, Monaghan, and Wexford) or the whole of Northern Ireland. Although the county and flock of origin of each sheep were recorded, it is probable that some of the sheep examined were sold between counties or flocks during their lifetimes. Exact official figures are not available, but it is estimated that approximately 56% of lambs go direct to slaughter from their farms of origin, 19% are sold for fattening and 25% are sold for breeding on other premises. It is unknown how many of the 19% of sheep sold for fattening or 25% sold for breeding either move between counties or remain in their county of origin (Personal communication; Caldwell, D., Sheep Identification and Movement Section, DAFM). Due to limited resources, only adult sheep were examined, as OPA occurs most frequently in this group. Furthermore, only lungs with gross lesions were collected for further analysis, therefore the true proportion of OPA-affected lungs may be underestimated as lungs with very small/microscopic lesions or latent JSRV infection would have been overlooked. Random sampling of sheep of any age and health status (i.e. including lambs and clinically-ill sheep) should be employed in future studies after careful statistical power calculations to determine the appropriate sample number to ascertain a more accurate prevalence for OPA. Information regarding the breed, sex, and exact age of the animals surveyed was not available. To the authors’ knowledge, no breed or sex predilections for OPA have been confirmed [1]. Although information regarding sex was not available, the vast majority of animals examined in the current study were cull ewes, while rams represented only a very small proportion of the study population. Therefore, the prevalence calculated in this study does not represent the entire Irish sheep population and should be considered as an estimate.

Disease status, age and geographical area all have an effect on the prevalence obtained in surveys for OPA. Lovatt and Strugnell (2013) investigated causes of death of ‘fallen ewes’ (ewes that died naturally on-farm) and found a relatively high percentage of OPA-positive animals (6.2%) reflecting that animals more likely to be clinically diseased were examined [31]. The current study focussed on cull animals, which, while considered clinically healthy on ante-mortem inspection, likely have health issues which are lead to loss of condition and hence culling. This may artificially increase the prevalence estimate obtained. Age also impacts on prevalence estimates as tumour formation is more likely to become apparent, both clinically and histologically, in adult animals, with disease most frequently evident in two to four-year-old sheep [1]. This study exclusively examined animals over one year old, thus increasing the chances of detecting OPA. Twenty-seven of the 31 OPA-positive sheep found in the 2015 UK survey) were recorded as adult animals, with the remaining four sheep classed as “age not recorded” (although these were thought likely to be adult sheep also) [18]. The geographical variation in OPA prevalence was shown by surveys in different parts of Iran where Khodakaram-Tafti and Razavi (2010) and Kajouri and Karimi (2002) found prevalences of 0.22% and 3%, respectively [19, 21].

The higher levels of OPA and JSRV infection in Donegal (8.9%, 15/168), the northernmost county in the Republic of Ireland which borders with Northern Ireland, compared to all the other counties, suggests a higher prevalence of JSRV and OPA in the north of the island of Ireland compared to the south. This is supported by data obtained from the All-Island Animal Disease Surveillance Reports which states that OPA is diagnosed more frequently in Northern Ireland [17]. For example, in 2015, OPA was the final diagnosis in 29% of sheep with respiratory disease necropsied by the Northern Ireland state veterinary laboratory compared to 3% of sheep with respiratory disease necropsied by DAFM in the Republic of Ireland [17]. However, it must be noted that all the animals from Donegal that tested positive for JSRV/OPA came from the same flock. Further testing would need to be carried out on other flocks originating from Donegal to ascertain if this is truly representative of the county as a whole, or simply an isolated, severely-affected flock. The positive results obtained from this flock greatly increased the overall countrywide prevalence estimate obtained, and possibly causes bias in the overall result.

Other factors that may affect the estimation of prevalence include the diagnostic tests used to identify infected animals. The use of different diagnostic tests and different target populations can yield very different prevalence estimates. For example, the use of PCR on blood from live sheep tends to yield higher JSRV prevalence estimates than do abattoir surveys using gross examination and histopathology. Lewis et al. (2011) used PCR on blood samples which resulted in a JSRV prevalence of 3.6% in sheep in Scotland [9], and even higher prevalence estimates of 18.75% and 18% were obtained in studies based in the central-west of Iran and northwest Iran, respectively, using PCR on blood samples [32, 33]. As PCR detects JSRV in circulating leukocytes, this technique can identify infection in latently and subclinically infected animals. However, because the sensitivity of this test is low (estimated at 11%) due to the low levels of infected cells (proviral DNA) in blood samples [9, 10], this method probably underestimates the true viral prevalence. Furthermore, it does not reflect the incidence of clinical disease, as most PCR-positive animals do not ultimately develop OPA [5].

The four cases of JSRV negative and IHC negative bronchiolo-alveolar adenocarcinoma identified in this study were unexpected and highlight the usefulness of the further diagnostic techniques employed in the present study. The lesions in these four cases were small, pale grey nodules, 0.5-2 cm in diameter. One had a soft core so was interpreted as an abscess. Three of these sheep came from flocks where no animals were positive for JSRV or OPA, while one of the four came from a flock in which one animal tested positive for JSRV by PCR only. The nodules were too small to cause respiratory disease, and would not be interpreted as fatal, end-stage OPA. If found incidentally at post-mortem examination however, they would probably have resulted in an erroneous diagnosis of early-stage OPA, based on histological examination alone. JSRV-negative bronchiolo-alveolar adenocarcinomas have been reported in New Zealand [22] and Australia [23], countries which are both officially free from OPA [1]. The New Zealand report stated that despite the millions of sheep lungs examined annually in abattoirs, no previous reports of lung tumours existed [22]. Interestingly, in the current study, examination of a comparatively small number of lungs from clinically normal sheep uncovered four such JSRV-negative bronchiolo-alveolar tumours, implying these lesions may be more common than previously thought.

In terms of diagnostic methodologies for OPA, a number of points are raised by this study. The majority of lungs (89%; 16/18) classified as MSO did not have histological lesions of OPA. The majority of these false-positive lesions (78%; 14/18) were due to chronic bronchopneumonia and 67% (12/18) were JSRV-negative by RT-PCR and IHC. Lesions of chronic bronchopneumonia in sheep are frequently locally-extensive, cranioventral in distribution, consolidated and grey-dark red in colour, so may mimic the gross appearance of OPA [25]. Our findings suggest follow-up histological examination of gross lesions suspicious of OPA is essential. This is already recognised as the confirmatory step in OPA diagnosis [13]. However, while animals examined in this study were considered clinically normal as they had passed ante-mortem examination, those that die of severe OPA presenting for post-mortem examination are likely to display much more extensive and grossly obvious lesions consistent with classical OPA (see caption of Image 1), and likely with clinical signs also. This would have been the case for the animals included in Table 1, where gross examination followed by histopathology was sufficient for diagnosis of OPA. Furthermore, the present study has demonstrated how OPA and bronchopneumonia can occur concurrently, with the pneumonic lesions obscuring the characteristic histological features of OPA, as was the case with one sheep in this study (Sheep 5). In another, chronic interstitial changes rendered tumour cells difficult to detect (Sheep 4). Cousens et al. (2015) also found that OPA-positive sheep frequently had concurrent abscesses, bronchopneumonia or fibrosis [18]. While the significance of these concurrent lesions with respect to susceptibility to OPA is unclear, retroviruses, such as JSRV, require dividing cells to replicate. Such cells would be present in greater numbers if there was a disease/ healing process ongoing in the lungs [5, 34]. Thus, in cases where OPA is strongly suspected but histological examination proves negative or inconclusive, follow-up IHC is advised. It was interesting to note that the PCR and IHC results were quite different, with many more sheep being positive by PCR than IHC, but that all sheep positive by IHC were also positive by PCR (except for one). In general, PCR is accepted as being a more sensitive diagnostic test than IHC. PCR detects viral nucleic acids, which are present in tumour cells and in circulating infected leukocytes [35]. Such leukocytes were likely present in low numbers in capillaries/lymphatics in lung sections taken for PCR. However, IHC detects viral proteins produced during viral translation and assembly, which occurs in OPA tumour cells. It has been shown that OPA cells are the only tissue in which JSRV can be reliably detected [36].

In the current study, 21 OPA lesion-free sheep were virus-positive by RT-PCR and 15 (71%) of these were from the flocks where cohorts had OPA lesions. These virus-positive sheep either represent latently-infected animals or those with microscopic lesions not present in the samples collected for histology. Similar findings were recorded by Caporale et al. (2005), Gonzalez et al. (2001) and Khodakaram Tafti et al. (2009) who found PCR-positive lungs of apparently tumour-free sheep that were in contact with OPA-positive sheep [5, 37, 38]. This supports the finding that in OPA-affected flocks many sheep are infected with JSRV and, for reasons that remain unclear, only a proportion develop OPA and clinical disease. Given the evidence that JSRV can be found more consistently in the lymphoreticular organs than lung tissue [5] future additional testing of pulmonary lymph nodes may assist in confirming JSRV infection in sheep in research studies or potentially in surveys/control programmes.

## Conclusions

This report confirms JSRV infection and OPA in sheep in the Republic of Ireland and provides estimated prevalence and geographical distribution data. Calculation of the true disease prevalence was beyond the scope of this study but the estimated prevalence is similar to that reported recently in the UK [18]. This work highlights the fact that in abattoir surveys, macroscopic examination of lungs alone is an insensitive way of determining the presence of OPA. The finding of non-viral bronchoalveolar adenomas highlights the need to further investigate single, small, well-demarcated neoplastic lung lesions in sheep using IHC and/or PCR.

## Additional file

Additional file 1: Table S2. Numbers of lesions of different types diagnosed by macroscopic examination. **Table S2**. Numbers of lesions of different types diagnosed by histologic examination. (DOCX 13 kb)

## Abbreviations

AFBI: Agri-Food and Biosciences Institute
BAL: Bronchoalveolar lavage
CI: Confidence Interval
CT: Cycle threshold
DAFM: Department of Agriculture, Food and the Marine
enJSRV: Endogenous JSRV-related retroviruses
H&E: Haematoxylin and eosin
HSO: Histologically-suspect OPA
IHC: Immunohistochemistry
JSRV: Jaagsiekte sheep retrovirus
MSO: Macroscopically-suspect OPA
NA: Nucleic acids
OPA: Ovine pulmonary adenocarcinoma
PBST: Phosphate buffered saline containing 0.05% Tween_20_
PCR: Polymerase chain reaction
QPCR: Quantitative PCR
RT-PCR: Reverse transcriptase polymerase chain reaction
UCD: University College Dublin
UK: United Kingdom

## Footnotes

1 €1 = UK£0.88 = $1.16 At time of writing
