# Supplementary material for: First confirmation by PCR of Jaagsiekte sheep retrovirus in Ireland and prevalence of ovine pulmonary adenocarcinoma in adult sheep at slaughter
Source: Ir Vet J. 2017 Dec 19;70:33. doi: 10.1186/s13620-017-0111-z (PMC5735933; doi:10.1186/s13620-017-0111-z)
Supplement: Additional file 1: Table S2. — Numbers of lesions of different types diagnosed by macroscopic examination. Table S2. Numbers of lesions of different types diagnosed by histologic examination. (DOCX 13 kb) [file 13620_2017_111_MOESM1_ESM.docx]

***Supplementary Tables***

Supplementary table 1: Numbers of lesions of different types diagnosed by macroscopic examination.

| **Macroscopic Lesion Category** | **Number of animals in which observed** | **% lungs sampled (n=369)** | **% lungs examined (n=1911)** |
| --- | --- | --- | --- |
| **Abscess(es)** | 74 | 20 | 3.9 |
| **Cranioventral consolidation** | 68 | 18.4 | 3.9 |
| **Discolouration** | 60 | 16.3 | 3.2 |
| **Fibrosis** | 56 | 15.2 | 2.9 |
| **Focal firm nodule** | 22 | 6.0 | 1.2 |
| **Mineralisation** | 13 | 3.5 | 0.7 |
| **Other** | 15 | 4.1 | 0.8 |
| **Subpleural parasitic granulomas** | 207 | 56.1 | 10.8 |
| **Macroscopic Suspect OPA (MSO)** | 18 | 4.8 | 0.9 |
| **Uncollapsed** | 12 | 3.3 | 0.6 |

Table 2: Numbers of lesions of different types diagnosed by histologic examination

| **Histological lesion category** | **Number of animals in which observed** | **% lungs sampled (n=369)** | **% lungs examined (n=1911)** |
| --- | --- | --- | --- |
| **Abscess** | 56 | 15.2 | 2.9 |
| **Bronchopneumonia** | 78 | 21.1 | 4.1 |
| **Interstitial thickening** | 74 | 20 | 3.0 |
| **Histologically Suspect OPA (HSO)** | 12 | 3.3 | 0.6 |
| **Parasitic infestation** | 148 | 40.1 | 7.7 |
| **Other** | 12 | 3.3 | 0.6 |
| **Normal** | 19 | 5.1 | 1.0 |
